# Gefitinib or erlotinib in previously treated non–small‐cell lung cancer patients: a cohort study in Taiwan

**DOI:** 10.1002/cam4.1121

**Published:** 2017-06-22

**Authors:** Chia‐Hao Chang, Chih‐Hsin Lee, Jen‐Chung Ko, Lih‐Yu Chang, Ming‐Chia Lee, Jann‐Yuan Wang, Chong‐Jen Yu

**Affiliations:** ^1^ Department of Internal Medicine National Taiwan University Hospital Hsinchu Branch Hsin Chu City Taiwan; ^2^ Division of Pulmonary Medicine Department of Internal Medicine Wan Fang Hospital Taipei Medical University Taipei Taiwan; ^3^ Division of Pulmonary Medicine Department of Internal Medicine School of Medicine College of Medicine Taipei Medical University Taipei Taiwan; ^4^ Department of Pharmacy New Taipei City Hospital New Taipei City Taiwan; ^5^ School of Pharmacy College of Pharmacy Taipei Medical University Taipei Taiwan; ^6^ Department of Internal Medicine National Taiwan University Hospital Taipei Taiwan

**Keywords:** chemotherapy, epidermal growth factor receptor‐tyrosine kinase inhibitor, non–small‐cell lung cancer, overall survival, progression‐free survival

## Abstract

Among treatment modalities for lung cancer, the most promising therapy is the use of epidermal growth factor receptor tyrosine kinase inhibitors (EGFR‐TKIs). Both erlotinib and gefitinib, the two first‐generation EGFR‐TKIs, exhibit significant clinical responses for patients with lung adenocarcinoma. However, few studies have compared the effects of these two drugs, and results have been inconclusive because of the small sample sizes in these studies. Therefore, this study was conducted to investigate this issue. This retrospective nationwide cohort study enrolled NSCLC patients who received EGFR‐TKIs after previous chemotherapy in Taiwan between 1996 and 2010 from the National Health Insurance Research Database. Clinical response and survival after receiving erlotinib and gefitinib were compared using logistic and Cox regression analyses, respectively. Inverse propensity score weighting and a sensitivity analysis in the EGFR‐TKI responder (clinical improvement by taking EGFR‐TKIs for 90 days), adherent patients (receiving EGFR‐TKI on a daily basis), adenocarcinoma, and adenocarcinoma with second‐line TKIs subgroup were performed for bias adjustment. A total of 7222 patients, including 4592 (63.6%) who received gefitinib, were identified. In the survival analysis, erlotinib was associated with a decline in 1‐year progression‐free survival (PFS) (hazard ratio, HR: 1.15 [1.09–1.21]) and overall survival (OS) (HR: 1.10 [1.03–1.18]). The effects of various TKIs were consistent in the 4939 EGFR‐TKI responders, adherent subgroup, adenocarcinoma subgroup, and adenocarcinoma with second‐line TKIs subgroup. In previously treated EGFT‐TKI‐naive NSCLC patients, those receiving gefitinib exhibited a longer PFS and OS than those receiving erlotinib. Additional large‐scale randomized controlled trials are warranted to confirm this finding.

## Introduction

Lung cancer is the leading cause of cancer‐related deaths worldwide 1. The treatment of advanced lung adenocarcinoma includes chemotherapy, target therapy, radiotherapy, and immunotherapy. Because epidermal growth factor receptor (EGFR) mutation accounts 11–22% of lung cancer driver mutations 2, 3, EGFR‐tyrosine kinase inhibitors (TKIs) have significantly improved the treatment outcome of lung cancer patients 4, 5. In previously treated non–small‐cell lung cancer (NSCLC) patients with unknown EGFR mutations status, erlotinib use was shown to significantly improve their overall survival (OS) compared with a placebo (median OS: 6.7 vs. 4.7 months; hazard ratio, HR: 0.70 [0.58–0.85]) 6. However, another study reported no difference in OS between gefitinib and placebo groups (median OS: 5.6 vs. 5.1 months; HR: 0.89 [0.77–1.02]) 7. A possible explanation for this finding is that the recommended dose of erlotinib (150 mg/day) was at the maximum tolerated dose (MTD) 7. Few prospective studies have compared the therapeutic response to various EGFR‐TKIs. In previously treated advanced lung adenocarcinoma patients, the PFS in the West Japan Oncology Group (WJOG) 5108L was similar in the gefitinib and erlotinib groups (6.5 vs. 7.5 months; HR: 1.125 [0.940–1.347]) 8. The efficacy of various EGFR‐TKIs remains debatable. The inconclusive results were attributed to the small sample size 8.

The practice of lung cancer treatment in Taiwan follows the National Comprehensive Cancer Network (NCCN) guideline. In 2009, the first‐line systemic therapy for advanced NSCLC is platinum‐based doublet chemotherapy and the second‐line therapy was single‐agent chemotherapy, erlotinib or gefitinib 9. In Taiwan before 2011, neither the molecular testing for the EGFR mutation was routinely performed nor EGFR‐TKIs were approved as first‐line therapy for advanced lung cancer in the National Health Insurance (NHI) program. The NHI approved gefitinib in 2004 and erlotinib in 2007 as second‐line therapy for pretreated lung adenocarcinoma and third‐line therapy for NSCLC.

The NHI program in Taiwan has provided mandatory universal health insurance with comprehensive medical care coverage since 1996, and it currently covers more than 99% of residents in Taiwan 10. With a longitudinal follow‐up of more than 22 million individuals, the National Health Insurance Research Database (NHIRD) is a very suitable data source for exploring the effects of an intervention in specific populations. Therefore, in this study, the NHIRD was employed to evaluate the difference in the efficacy of erlotinib and gefitinib in previously treated NSCLC patients.

## Materials and Methods

The Institutional Review Board of National Taiwan University Hospital approved the study (NTUH REC: 201212001W) and waived the need for informed consent because of the retrospective design and use of an encrypted database.

### Case selection

Lung cancer patients were selected using a compatible diagnosis (International Classification of Disease, 9th Revision, Clinical Modification [ICD‐9‐CM] code 162) from the Registry of Catastrophic Illness Patients Database, a subset of the NHIRD. To apply to this registry, histological confirmation is obligatory. The index date was defined as the date that patients applied to this registry for lung cancer.

The NHIRD was searched for key chemotherapy drugs for NSCLC according to the National Comprehensive Cancer Network Guidelines for NSCLC; the search included gemcitabine, vinorelbine, docetaxel, paclitaxel, etoposide, and pemetrexed 11. First‐line chemotherapy was defined as the first key chemotherapy drug used after the index date. The start and end dates of first‐line chemotherapy were identified.

The EGFR‐TKIs investigated in this study were gefitinib and erlotinib. Afatinib had not yet been approved by NHI during the study period. The prescription duration of individual EGFR‐TKIs was converted from the claims data according to the defined daily doses (DDDs) 12. These drugs required preaudit approval by the NHI administration; they benefit patients with lung adenocarcinoma only as a second‐line therapy (after failure of first‐line chemotherapy) and patients with NSCLC as a third‐line therapy. Approval is reaudited every 90 days and reissued only to patients with treatment responses to EGFR‐TKIs according to the criteria of the Response Evaluation Criteria in Solid Tumors (RECIST) group (i.e., stable disease or partial or complete response) 13. In this study, patients who discontinued EGFR‐TKIs within 90 days were classified as EGFR‐TKI nonresponders, and others were classified as EGFR‐TKI responders.

**Figure 1:**
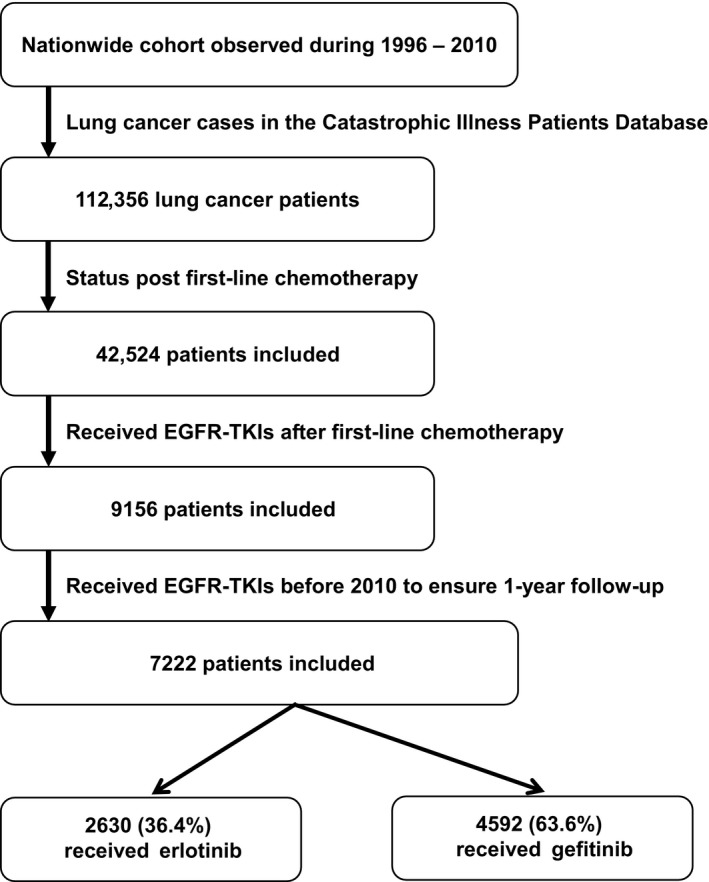
Selection and disposition of the study subjects. EGFR‐TKIs, epidermal growth factor receptor‐tyrosine kinase inhibitors.

Patients who started EGFR‐TKIs earlier than the end date of first‐line chemotherapy were excluded. Patients who first received EGFR‐TKIs in 2010 or later were also excluded to ensure at least 1 year of follow‐up. The complete selection process is presented in Figure 1.

### Comorbidities

Comorbidities such as chronic obstructive pulmonary disease (COPD), diabetes mellitus, chronic kidney disease (CKD), liver cirrhosis, pneumoconiosis, autoimmune disease, organ transplantation, AIDS, and other malignancies were identified according to a previous study 14. Insurance status was used to identify low‐income earners, the threshold of which was defined as an annual household income of <US$4500.

### Disease severity

We used several surrogates between the index date and start date of EGFR‐TKI use to adjust for disease severity between gefitinib and erlotinib for possible selection bias by primary physicians. These surrogates, including cachexia 15, intracranial metastasis 16, anemia 17, and duration of hospitalization (days), are strong predictors of low cancer survival. Patients were defined as having cachexia if they had previously used megestrol or medroxyprogesterone. Intracranial metastasis was defined according to patients exhibited increased intracranial pressure (IICP), which was determined by whether they had been prescribed glycerin or mannitol. Anemia was defined as requiring transfusion of packed red blood cells (PRBCs). Surgery was defined as ever having received pulmonary wedge resection, segmental resection, lobectomy, or pneumonectomy. Patients who had received surgery implied earlier cancer staging with longer survival.

### Statistical analysis

For previously treated EGFT‐TKI‐naive NSCLC patients and the EGFR‐TKI responders among them, intergroup differences were compared using the *t* test or Mann–Whitney *U* test for continuous variables on the basis of their normality, and the chi‐squared test or Fisher's exact test was used for categorical variables, as appropriate. For each variable, 1‐year PFS and 1‐year OS (both from the start of EGFR‐TKI use) were generated using the Kaplan–Meier method and compared using the log‐rank test. Cox proportional hazards regression analysis was performed to identify the independent prognostic factors.

We derived a propensity score, which is the logit (probability) for receiving erlotinib or gefitinib treatment from a multinomial logistic regression model by using crucial background covariates, including age, gender, operation, cachexia, IICP, PRBC transfusion, duration of hospitalization (days), COPD, diabetic mellitus, CKD, other malignancy, autoimmune disease, liver cirrhosis, transplantation, AIDS, and low income. Inverse propensity score weighting (IPSW) was used in the Cox model to adjust for potential confounders in selecting erlotinib and gefitinib 18.

In the multivariate analysis, potential interactions between variables were checked, and all variables were included. Statistical significance was set at *P *< 0.05. All analyses were conducted using SPSS version 20.0 (SPSS Inc., Chicago, IL), except the IPSW, which was performed using R version 3.3.1 (R Foundation for Statistical Computing, Vienna, Austria).

### Sensitivity analysis

Sensitivity analysis was performed to evaluate the impact of erlotinib versus gefitinib in three subgroups: EGFR‐TKI adherent population, histological adenocarcinoma, and second‐line TKIs for adenocarcinoma.

Adherence to EGFR‐TKI was assessed by calculating the total DDD of EGFR‐TKIs divided by the duration between the start and end dates of EGFR‐TKI use (medication possession ratio) 19. Patients were considered adherent if they received EGFR‐TKI on a daily basis. Patients were assumed to have histological adenocarcinoma if they had previously received pemetrexed, which requires preapproval by the NHI administration with histological evidence of adenocarcinoma.

## Results

### Patient selection

For the 1996–2010 period, a total of 112,356 lung cancer patients were identified; 42,524 of them had previously received first‐line chemotherapy. Among these patients, 9156 patients were prescribed erlotinib or gefitinib after first‐line chemotherapy. Among them, 1934 patients received EGFR‐TKIs after 2009 and were excluded from the analysis. The final sample comprised 7222 patients; 4592 (63.6%) received gefitinib and 2630 (36.4%) received erlotinib (Fig. 1). Among the 7222 patients, 4939 were TKI responders, including 3278 who received gelfitinib.

Table 1 present a comparison of the demographic data between the erlotinib and gefitinib groups. The mean age of the entire sample was 61.0 ± 12.0, with 40.9% of the sample aged 65 years or older, and 49.4% of the patients were men. Drug adherence was 98.3% on average. In terms of disease severity, cachexia, IICP, and a history of operation were noted in 38.1%, 34.1%, and 11.0% of the patients, respectively. The mean duration of hospitalization was 26.5 days between the index date and start date of EGFR‐TKI use, and the mean unit of PRBC transfusion was 1.1. The most common underlying comorbidities were diabetes mellitus (22.3%), COPD (15.1%), and malignancies other than lung cancer (8.4%).

**Table 1:** Patient characteristics based on different epidermal growth factor receptor tyrosine kinase inhibitors (EGFR‐TKIs)

Variables	Overall		EGFR‐TKI responder^a^	
	Erlotinib (*n *= 2630)	Gefitinib (*n *= 4592)	Erlotinib (*n *= 1661)	Gefitinib (*n *= 3278)
Male	1651 (62.8)^b^	1915 (41.7)	971 (58.5)^b^	1250 (38.1)
Age (years)	61.7 ± 11.9^b^	60.6 ± 12.0	61.3 ± 11.9	60.6 ± 11.9
≥65	1135 (43.2)^b^	1820 (39.6)	687 (41.4)	1288 (39.3)
Adenocarcinoma^c^	966 (36.7)^b^	1512 (32.9)	617 (37.1)	1141 (34.8)
Disease severity				
Operation	284 (10.8)	511 (11.1)	193 (11.6)	397 (12.1)
Cachexia	1063 (40.4)^b^	1692 (36.8)	614 (37.0)	1154 (35.2)
Increased intracranial pressure	952 (36.2)^b^	1514 (33.0)	622 (37.4)^b^	1077 (32.9)
Duration of hospitalization (days)	25.0 ± 37.8^b^	27.3 ± 43.0	23.8 ± 41.0^b^	26.8 ± 46.2
PRBC transfusion (unit)	1.2 ± 2.5	1.1 ± 2.3	1.0 ± 2.4	1.0 ± 2.2
Comorbidity				
Diabetes mellitus	593 (22.5)	1014 (22.1)	372 (22.4)	728 (22.2)
COPD	503 (19.1)^b^	586 (12.8)	295 (17.8)^b^	388 (11.8)
Other malignancies	221 (8.4)	384 (8.4)	129 (7.8)	274 (8.4)
Autoimmune disease	10 (0.4)^b^	36 (0.8)	7 (0.4)	23 (0.7)
End‐stage renal disease	8 (0.3)	17 (0.4)	4 (0.2)	10 (0.3)
Chronic kidney disease	158 (6.0)	253 (5.5)	96 (5.8)	184 (5.6)
Liver cirrhosis	3 (0.1)^b^	0 (0)	1 (0.1)	0 (0)
Transplantation	0 (0)	2 (0.04)	0 (0)	2 (0.1)
AIDS	0 (0)	1 (0.02)	0 (0)	1 (0.03)
Low income	67 (2.5)^b^	75 (1.6)	44 (2.6)^b^	52 (1.6)
Drug adherence^d^ (%)	98.9 ± 19.9^b^	97.3 ± 22.8	92.1 ± 17.8^b^	95.0 ±15.3
EGFR‐TKI responder^a^	1661 (63.2)^b^	3278 (71.4)	N/A	N/A

AIDS, acquired immunodeficiency syndrome; COPD, chronic obstructive pulmonary disease; PRBCs, packed red blood cells.

Data are presented as number (%) or mean ± standard deviation.

a Patients who received EGFR‐TKIs for more than 90 days.

b *P* < 0.05 between the erlotinib and gelfitinib groups.

c Patients who previously received pemetrexed.

d Drug adherence was noted by calculating the medication possession ratio of EGFR‐TKIs.

In the whole cohort, the erlotinib group mostly comprised men (62.8% vs. 41.7%, *P *< 0.001) who were older (61.7 vs. 60.6 years, *P *< 0.001) and had cachexia (40.4% vs. 36.8%, *P *= 0.003), IICP (36.2% vs. 33.0%, *P *= 0.005), a shorter duration of hospitalization (25.0 vs. 27.3 days, *P *= 0.015), COPD (19.1% vs. 12.8%, *P *< 0.001), and higher drug adherence (98.9% vs. 97.3%, *P *= 0.002). The findings were similar in the EGFR‐TKI responders (Table 1). Among them, the erlotinib group mostly comprised men (58.5% vs. 38.1%, *P *< 0.001) and had IICP (37.4% vs. 33.9%, *P *= 0.001), a shorter duration of hospitalization (23.8 vs. 26.8 days, *P *= 0.019), COPD (17.8% vs. 11.8%, *P *< 0.001), and lower drug adherence (92.1% vs. 95.0%, *P *< 0.001).

### Propensity score of EGFR‐TKIs

Logistic regression revealed that patients were more likely to receive erlotinib if they were men (odds ratio, OR: 2.36 [2.14–2.60]), older (OR: 1.01 [1.00–1.01] for per‐year increment in age), had cachexia (OR: 1.16 [1.05–1.28]), IICP (OR: 1.15 [1.04–1.28]), and were low‐income earners (OR: 1.57 [1.13–2.20]). By contrast, patients with longer duration of hospitalization (OR: 0.998 [0.997–1.000]) and autoimmune disease (OR: 0.48 [0.24–0.98]) were more likely to use gefitinib. All these factors were included in the propensity score calculation.

### Prognostic factors of 1‐Year PFS

**Figure 2:**
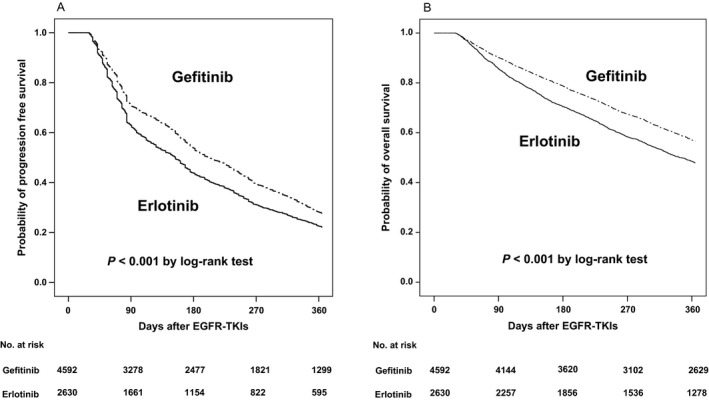
Kaplan–Meier curves for (A) 1‐year progression‐free survival in erlotinib versus gefitinib and (B) 1‐year overall survival in erlotinib versus gefitinib.

In whole cohort, the Kaplan–Meier analysis revealed that the erlotinib group had a poorer 1‐year PFS than the gefitinib group (median: 5.1 vs. 6.8 months; Fig. 2A). The results of the multivariate Cox regression analysis before and after IPSW adjustment for 1‐year PFS were similar. Besides EGFR‐TKI, poor prognostic factors included male gender, age ≥ 65 years, cachexia, longer duration of hospitalization, and PRBC transfusion (Table 2, left panel). Patients who had previously received pulmonary surgery exhibited a more favorable prognosis.

**Table 2:** Multivariate Cox proportional hazards regression analysis for 1‐year progression‐free survival with inverse propensity score weighting (IPSW) adjustment

Variable	Whole cohort		EGFR‐TKI responder	
	No adjustment	IPSW	No adjustment	IPSW
Erlotinib	1.17 (1.11–1.24)	1.15 (1.09–1.21)	1.11 (1.03–1.20)	1.11 (1.03–1.17)
Male	1.35 (1.27–1.42)	1.38 (1.32–1.44)	1.22 (1.14–1.32)	1.26 (1.18–1.34)
Disease severity				
Operation	0.73 (0.67–0.80)	0.75 (0.69–0.80)	0.71 (0.63–0.80)	0.71 (0.64–0.78)
Cachexia	1.20 (1.13–1.27)	1.24 (1.19–1.30)	1.15 (1.07–1.24)	1.19 (1.12–1.27)
Duration of hospitalization (days)	1.001 (1.000–1.001)	1.001 (1.000–1.001)	1.001 (1.000–1.001)	1.001 (1.001–1.0014)
Packed red blood cell transfusion	1.03 (1.02–1.04)	1.04 (1.03–1.04)	1.03 (1.14–1.32)	1.03 (1.02–1.04)

Multivariate Cox regression adjusted for gender, age, disease severity (operation, cachexia, increased intracranial pressure, duration of hospitalization [days], and transfusion), comorbidities (chronic obstructive pulmonary disease, diabetic mellitus, chronic kidney disease, tuberculosis, liver cirrhosis, autoimmune disease, transplantation, acquired immunodeficiency syndrome, and other malignancies), and low income.

Data were hazards ratio and 95% confidence interval.

EGFR‐TKI, epidermal growth factor receptor‐tyrosine kinase inhibitors.

The results of analyses in the EGFR‐TKI responders were similar as those in the whole cohort. The median PFS for erlotinib recipients was 8.9 months and that for gefitinib recipients was 10.2 months. In the multivariate Cox regression analysis with IPSW adjustment, erlotinib was independently associated with a poorer 1‐year PFS than gefitinib (HR: 1.11 [1.03–1.17]) (Table 2, right panel). Other independent poor prognostic factors included male gender (HR: 1.26 [1.18–1.34]), cachexia (HR: 1.19 [1.12–1.27]), longer duration of hospitalization (HR: 1.001 [1.001–1.0014]), and PRBC transfusion (HR: 1.03 [1.02–1.04]). However, patients who had previously received pulmonary surgery were associated with a more favorable 1‐year PFS (HR: 0.71 [0.64–0.78]).

### Prognostic factors of 1‐year OS

In the whole cohort, the Kaplan–Meier analysis revealed that the erlotinib group had a poorer 1‐year OS than the gefitinib group (median: 11.5 vs. 13.8 months; Fig. 2B). The results of the multivariate Cox regression analysis before and after IPSW adjustment for 1‐year OS were similar. Besides EGFR‐TKI, poor prognostic factors included male gender, age ≥ 65 years, cachexia, IICP, and PRBC transfusion (Table 3, left panel). Patients who had previously received pulmonary surgery or were EGFR‐TKI responders exhibited a more favorable prognosis.

**Table 3:** Multivariate Cox proportional hazards regression analysis for 1‐year overall survival with inverse propensity score weighting (IPSW) adjustment

Variable	Whole cohort		EGFR‐TKI responder	
	No adjustment	IPSW	No adjustment	IPSW
Erlotinib	1.32 (1.23–1.41)	1.10 (1.03–1.18)	1.22 (1.14–1.31)	1.08 (0.98–1.18)
Male	1.54 (1.44–1.65)	1.37 (1.29–1.45)	1.48 (1.38–1.60)	1.52 (1.40–1.65)
Age ≥ 65 years	1.17 (1.09–1.25)	1.18 (1.11–1.25)	1.09 (1.02–1.17)	1.22 (1.12–1.33)
EGFR‐TKI responder	0.22 (0.20–0.23)	0.21 (0.20–0.23)	N/A	N/A
Disease severity				
Operation	0.59 (0.52–0.67)	0.63 (0.57–0.70)	0.59 (0.52–0.67)	0.63 (0.55–0.74)
Cachexia	1.37 (1.28–1.47)	1.26 (1.19–1.34)	1.30 (1.22–1.39)	1.40 (1.28–1.52)
Increased intracranial pressure	1.14 (1.06–1.22)	1.29 (1.21–1.36)	1.16 (1.08–1.25)	1.30 (1.19–1.42)
Duration of hospitalization (days)	1.001 (1.000–1.002)		1.001 (1.001–1.002)	
Packed red blood cell transfusion	1.07 (1.06–1.08)	1.08 (1.07–1.09)	1.06 (1.05–1.07)	1.07 (1.05–1.08)

Multivariate Cox regression adjusted for gender, age, disease severity (operation, cachexia, increased intracranial pressure, duration of hospitalization [days], and transfusion), comorbidities (chronic obstructive pulmonary disease, diabetes mellitus, chronic kidney disease, tuberculosis, liver cirrhosis, autoimmune disease, transplantation, AIDS, and other malignancies), EGFR‐TKI responder, and low income.

Data were hazards ratio and 95% confidence interval.

EGFR‐TKIs, epidermal growth factor receptor‐tyrosine kinase inhibitors.

The results of analyses in the TKI responders were similar as those in the whole cohort, except that erlotinib was not associated with a poorer 1‐year OS than gefitinib after IPSW adjustment (HR: 1.08 [0.98–1.18]) (Table 3, right panel). The median OS for erlotinib recipients was 14.9 months and that for gefitinib recipients was 16.5 months. Other independent factors of a poor prognosis included male gender (HR: 1.52 [1.40–1.65]), age ≥ 65 years (HR: 1.22 [1.12–1.33]), cachexia (HR: 1.40 [1.28–1.52]), IICP (HR: 1.30 [1.19–1.42]), and PRBC transfusion (HR: 1.07 [1.05–1.08]). However, patients who had previously received pulmonary surgery exhibited a more favorable prognosis (HR: 0.63 [0.55–0.74]).

### Sensitivity analysis

The results of the sensitivity analysis performed using multivariate Cox analysis with IPSW adjustment were consistent with those of the whole cohort and the EGFR‐TKI responders (Table 4), indicating that having received erlotinib was an independent factor of a poor prognosis in all three subgroups. The HR for 1‐year PFS for receiving erlotinib was 1.15 (1.09–1.21) in the whole cohort, 1.11 (1.03–1.17) in the EGFR‐TKI responders, 1.16 (1.08–1.25) in the adherent subgroup, 1.35 (1.24–1.47) in the adenocarcinoma subgroup, and 1.39 (1.22–1.59) in the adenocarcinoma with second‐line TKIs subgroup. The HR for 1‐year OS for erlotinib recipients was 1.10 (1.03–1.18) in the whole cohort, 1.08 (0.98–1.18) in the EGFR‐TKI responders, 1.08 (1.02–1.16) in the adherent subgroup, 1.89 (1.62–2.09) in the adenocarcinoma subgroup, and 1.87 (1.47–2.37) in the adenocarcinoma with second‐line TKIs subgroup (Table 4).

**Table 4:** Impact of Erlotinib versus Gefitinib on 1‐year progression‐free survival and 1‐year overall survival in the whole cohort, epidermal growth factor receptor‐tyrosine kinase inhibitor (EGFR‐TKI) responder, and three different subgroups by multivariate Cox proportional hazards regression analysis with inverse propensity score weighting adjustment

Erlotinib vs. Gefitinib	1‐year progression‐free survival			1‐year overall survival		
	HR	95% CI	*P* value	HR	95% CI	*P* value
Whole cohort (*n *= 7222)	1.15	1.09–1.21	<0.001	1.10	1.03–1.18	0.003
EGFR‐TKI responder^a^^,^ c(*n *= 4939)	1.11	1.03–1.17	0.006	1.08	0.98–1.18	0.122
Adherent population^b^ (*n *= 4079)	1.09	1.02–1.16	0.010	1.08	1.02–1.16	0.030
Adenocarcinoma^c^ (*n *= 2478)	1.35	1.24–1.47	<0.001	1.89	1.62–2.19	<0.001
Second‐line, adenocarcinoma^c^ (*n *= 1181)	1.39	1.22–1.59	<0.001	1.87	1.47–2.37	<0.001

Multivariate Cox regression adjusted for gender, age, disease severity (operation, cachexia, increased intracranial pressure, duration of hospitalization [days], and transfusion), comorbidities (chronic obstructive pulmonary disease, diabetes mellitus, chronic kidney disease, tuberculosis, liver cirrhosis, autoimmune disease, transplantation, AIDS, and other malignancies), EGFR‐TKI responder, and low income.

a Patients who received epidermal growth factor receptor‐tyrosine kinase inhibitors (EGFR‐TKIs) for more than 90 days.

b Patients with a medication possession ratio of EGFR‐TKIs ≥ 1.

c Patients who previously received pemetrexed.

## Discussion

This large retrospective cohort study used NHIRD to compare the outcome of two first‐generation EGFR‐TKIs, erlotinib and gefitinib. Three major findings were obtained. First, in previously treated lung cancer patients, gefitinib independently provided more favorable 1‐year PFS and OS compared with erlotinib. Moreover, the benefit was observed in four subpopulations: EGFR‐TKI responders, adherent patients, adenocarcinoma patients, and adenocarcinoma patients receiving TKIs as second‐line therapy. Second, male gender, cachexia, longer duration of hospitalization, and PRBC transfusion were associated with poorer survival. Third, erlotinib was more likely to be prescribed to patients with higher disease severity, such as those with cachexia and IICP.

The first‐generation EGFR‐TKIs, gefitinib and erlotinib, are reversible inhibitors. These drugs have been extensively evaluated for NSCLC treatment. In the BR.21 trial, patients who previously received chemotherapy and then erlotinib demonstrated a significant OS benefit compared with those who received a placebo (median OS: 6.7 vs. 4.7 months; HR: 0.70 [0.58–0.85]) 6. However, in the Iressa Survival Evaluation in Lung Cancer (ISEL) trial, which had a similar study design, gefitinib demonstrated no difference in OS versus placebo (median OS: 5.6 vs. 5.1 months; HR: 0.89 [0.77–1.02]) 7. On the basis of these two studies, erlotinib appears to be more effective than gefitinib. However, compared with chemotherapy, both erlotinib and gefitinib have been shown to demonstrate noninferiority in PFS 20, 21, 22. These trials enrolled a mixed population of patients with and without EGFR mutations. Moreover, in the TAILOR trial, which compared docetaxel with erlotinib as a second‐line treatment in NSCLC without EGFR mutations in exons 19 and 21, docetaxel use was shown to benefit survival 23. Therefore, the EGFR mutation status remains crucial beyond first‐line therapy in NSCLC patients.

Three retrospective studies have compared erlotinib with gefitinib beyond first‐line therapy in NSCLC patients, all of which reported similar efficacy and outcomes between erlotinib and gefitinib, regardless of EGFR status 24, 25, 26. Among NSCLC patients, irrespective of EGFR mutation status, median PFS and OS have been shown to be 4.6–5.0 and 12.6–18 months under gefitinib treatment, and 2.7–2.9 and 11.6–12.1 months under erlotinib treatment, respectively 24, 25. In another study, patients harboring sensitive EGFR mutation exhibited a PFS of 11.7 and 9.6 months for gefitinib and erlotinib, respectively 26. Although statistically nonsignificant, the results revealed longer median PFS and OS in gefitinib recipients 24, 25, 26. Recently, Urata et al. reported a randomized phase III study, the WJOG 5108L, which did not demonstrate noninferiority between gefitinib and erlotinib in PFS (6.5 vs. 7.5 months; HR: 1.125 [0.940–1.347]) and OS (22.8 vs. 24.5 months; HR: 1.038 [0.833–1.294]) in patients with previously treated advanced lung adenocarcinoma. The median PFS in patients harboring activating EGFR mutation in the gefitinib and erlotinib groups was also similar (8.3 vs. 10.0 months; HR: 1.093 [0.879–1.358]) 8.

Because a larger cohort was used, this study demonstrated a significantly higher 1‐year PFS and OS in patients receiving gefitinib compared with those receiving erlotinib. The results were consistent after adjusting for disease severity, comorbidities, and factors affecting the decision of selecting either erlotinib or gefitinib. Further analyses revealed that gefitinib use remained a strong predictor for longer PFS and OS in EGFR‐TKI responders, adherent patients, adenocarcinoma patients, and adenocarcinoma patients receiving EGFR‐TKIs as second‐line therapy. In contrast to previous studies that used smaller samples (242–716 patients) 24, 25, 26, statistical significance was achieved in this study mainly because of the high number of cases identified from the NHIRD. Although in clinical practice, erlotinib is the more preferred drug among primary care physicians for patients with cachexia, IICP, COPD, and male gender, the results of this study show that the survival benefit from gefitinib was significantly larger than that from erlotinib after adjusting for these factors and applying IPSW in the multivariate Cox regression analysis.

Several possible explanations can be offered for the superior therapeutic effect of gefitinib. First, the MTD was selected for erlotinib (150 mg/day) but not for gefitinib (250 mg/day) 27, 28. The area under the concentration–time curve showed interpatient variability and increased linearly with once‐daily dosing ranging from 10 to 100 mg in gefitinib and 25 to 200 mg in erlotinib 29. This indicates that a higher dose of gefitinib (500 mg/day) would be less effective and more toxic. Thus, gefitinib could achieve the maximal anticancer effect without using the MTD (800 mg) 30. Second, gefitinib has a higher volume of distribution (1700 L)^31^ than erlotinib (233 L) 32. The high distribution volume of gefitinib implies significant passage of the drug out of the circulation and into the tissues 33. A clinical study of NSCLC patients confirmed that the tumor tissue concentration of gefitinib is markedly higher than the plasma concentration 34. By contrast, the tumor/plasma concentration ratio of erlotinib was approximately 63% in a clinical study of lung cancer 35. Thus, gefitinib could reach tumor cells more efficiently than erlotinib. Third, gefitinib has a longer half‐life (41 h) than erlotinib (36 h) 33, which ensures that the gefitinib concentration remains consistent. Fourth, erlotinib, but not gefitinib metabolism, partially depends on cytochrome P450 (CYP) 1A1 and CYP1A2 36. Smoking is known to increase CYP1A2 and CYP1A1 activity, thus increasing clearance and reducing the efficacy of erlotinib 37.

This study has some limitations. First, information on the initial staging of lung cancer was not available. Although we used pulmonary surgery as a surrogate for early‐stage lung cancer and the survival difference between erlotinib and gefitinib remained significant after adjusting for this factor, some bias may have remained. Second, results of molecular testing for EGFR gene mutation were not available in the cohort. Because the approval of EGFR‐TKI use for > 90 days in Taiwan requires a reaudit based on the therapeutic response, we used the prescription duration of TKI > 90 days as a marker for sensitive EGFR gene mutation. The advantage of gefitinib remained in the whole cohort, EGFR‐TKI responders, and the sensitivity analysis. Third, the performance status of each patient was unavailable. Therefore, the presence of cachexia, IICP, red blood cell transfusion, and longer duration of hospitalization were used as surrogates for disease severity. Nevertheless, bias may have still affected the results. Lastly, because the EGFR mutation rate is different between white and Asian populations 38, several large randomized lung cancer studies investigating EGFR‐TKI therapy included only Asian populations 4, 8. In these studies, the PFS and OS were similar as those obtained in this study 24, 25, 26. Therefore, the results of this study may apply to all Asian but not non‐Asian population.

## Conclusion

The result of this study provides evidence that the efficacy of two first‐generation EFGR‐TKIs varies. Gefitinib appears to be more effective than erlotinib in previously treated NSCLC patients. Additional prospective, large‐scale, randomized control trials are necessary to confirm this finding.

## Conflicts of Interest

The authors have no conflicts of interests to declare.
